# Physiological Signal Analysis and Stress Classification from VR Simulations Using Decision Tree Methods

**DOI:** 10.3390/bioengineering10070766

**Published:** 2023-06-26

**Authors:** Syem Ishaque, Naimul Khan, Sridhar Krishnan

**Affiliations:** Department of Electrical, Computer and Biomedical Engineering, Toronto Metropolitan University, Toronto, ON M5B 2K3, Canada; n77khan@torontomu.ca

**Keywords:** VR video game, personalized CART model, K-means feature, Gini index, stress, HRV

## Abstract

Stress is induced in response to any mental, physical or emotional change associated with our daily experiences. While short term stress can be quite beneficial, prolonged stress is detrimental to the heart, muscle tissues and immune system. In order to be proactive against these symptoms, it is important to assess the impact of stress due to various activities, which is initially determined through the change in the sympathetic (SNS) and parasympathetic (PNS) nervous systems. After acquiring physiological data wirelessly through captive electrocardiogram (ECG), galvanic skin response (GSR) and respiration (RESP) sensors, 21 time, frequency, nonlinear, GSR and respiration features were manually extracted from 15 subjects ensuing a baseline phase, virtual reality (VR) roller coaster simulation, color Stroop task and VR Bubble Bloom game. This paper presents a comprehensive physiological analysis of stress from an experiment involving a VR video game Bubble Bloom to manage stress levels. A personalized classification and regression tree (CART) model was developed using a novel Gini index algorithm in order to effectively classify binary classes of stress. A novel K-means feature was derived from 11 other features and used as an input in the Decision Tree (DT) algorithm, strong learners Ensemble Gradient Boosting (EGB) and Extreme Gradient Boosting (XGBoost (XGB)) embedded in a pipeline to classify 5 classes of stress. Results obtained indicate that heart rate (HR), approximate entropy (ApEN), low frequency and high frequency ratio (LF/HF), low frequency (LF), standard deviation (SD1), GSR and RESP all reduced and high frequency (HF) increased following the VR Bubble Bloom game phase. The personalized CART model was able to classify binary stress with 87.75% accuracy. It proved to be more effective than other related studies. EGB was able to classify binary stress with 100% accuracy, which outperformed every other related study. XGBoost and DT were able to classify five classes of stress with 72.22% using the novel K-means feature. This feature produced less error and better model performance in comparison to using all the features. Results substantiate that our proposed methods were more effective for stress classification than most related studies.

## 1. Introduction

**Figure 1:**
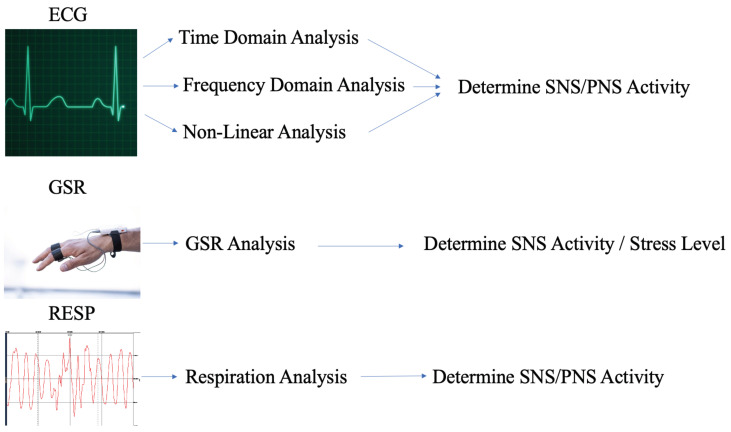
Process to retrieve SNS and PNS activty information from each sensor.

Physiological stress is considered one of the most common causes of several health problems occurring throughout the world. Heart Rate Variability (HRV) is habitually utilized for effective computing, cognitive workload and stress analysis [1]. It is an effective way to measure the rate of change associated with each heartbeat. This rate of change is controlled by the Autonomic nervous system (ANS), more specifically its two subcomponents: the Sympathetic nervous system (SNS) and Parasympathetic nervous system (PNS). The Hypothalamus receives information from the two branches of the ANS and signals the rest of the body to stimulate or relax different functions in order to maintain homeostasis. SNS activity increases and PNS activity decreases in response to increased stress. An electrocardiogram (ECG) is a simple and non invasive technique to measure R-R intervals associated with each heartbeat. Variations within each interval between consecutive heartbeats represent HRV. SNS activity reduces the variation between each R-R interval (the interval between the two consecutive R-waves in an ECG), leading to an increased heart rate (HR), reduced HRV and increased stress [2]. PNS activity alternatively increases the variation between each R-R interval, which increases HRV, leads to relaxation and decreases the HR. A respiration (RESP) signal is used to measure the respiration rate, which shares a high correlation with HRV and PNS activity. It significantly increases typically above 25 breaths/min during stressful situations [3]. Galvanic skin response (GSR) is used to measure the rate of change associated with electrodermal activity (EDA). Skin temperature changes when a person is aroused or excited, which causes a change in electrical conductance [4]. GSR is also referred to as EDA or Skin Conductance (SC). It refers to the variation in sweat gland activity due to emotional arousal. EDA represents the variation in electrical conductance of the skin due to sweat secretion. Sweat secretion causes a change within the balance of positive and negative ions which allows electrical current to flow more effortlessly. This results in changing the skin conductance. There is a decrease in resistance and an increase in electrical conductance. This activity results in increased sweat gland activity and indicates that there is an increase in arousal in response to the active situation experienced from the environment, which produced an emotional change or response. Generally, dry skin has a much higher skin resistance than wet skin, so when someone is stressed, they will likely secrete more sweat gland and reduce skin resistance, resulting in a warmer skin or vivid sweat. Our emotional response would be something intense, as a result of the increased activity and skin conductance. GSR ideally represents the variation associated with electrical characteristics of the skin. An increase in stress levels impacts sweat gland activity, which is regulated by the SNS activity. GSR is mainly effective for measuring the increase in stress levels and increased SNS activity. Figure 1 illustrates the protocol for SNS and PNS activity data acquisition through ECG, GSR and RESP sensors.

Video games have recently gained recognition for their ability to improve social skills, stimulate mental activity and increase stress resilience [5]. Aliyary et al. studied the beneficial and harmful effects of video games on cognitive functions [6]. They analyzed the function of alpha-amylase, cortisol and brain waves due to stress using electroencephalogram (EEG) signals. The results indicate that depending on the type of video games, the stress level varies as well. Non-violent games that can stimulate mental activity, such as Tetris, are beneficial for stress levels. Roy et al. studied the impact of competitive and cooperative video games on stress levels [7]. The results obtained from HR, blood pressure (BP) and a self-reported questionnaire on stress and valence indicate that video games can improve social skills and reduce stress. The group conveyed the message that video games are a great way to socialize with one and another, play cooperatively and distract people from stress. Reinecke et al. conducted an experiment to analyze whether strain is an effective way to recuperate from work stress [8]. Upon analyzing the frequency of game use, recovery time, relaxation and daily hassle, they came to the conclusion that video games can in fact help people recover after being exposed to stressful situations. Research using video games is steadily increasing, but machine learning and physiological signal analysis within this domain is limited. HRV analysis in the frequency domain is the most effective method of HRV analysis. It generally comprises a very low frequency (VLF) (0.003–0.04 Hz) band, a low frequency (LF) (0.04–0.15 Hz) band and a high frequency (HF) (0.015–0.4 Hz) band [2]. LF is predominantly used to study the ramifications of SNS activity but can also analyze PNS activity associated with respiration and HF comprehensively delineates PNS activity [9]. Rosenberg et al. studied the LF/HF ratio through 2D scatter plots in order to analyze physiological and psychological states from various stressful scenarios [2]. They determined that scatter plots were a more effective measure of stress rather than standard LF/HF ratio. They did not analyze stress variations with respect to time. Hsu et al. analyzed the correlation between non-linear standard deviation (SD1), (SD2), Poincaré approximate entropy (ApEn) and power spectral density (PSD) in a frequency domain, using data obtained from anesthesia [10]. SD1 represents the standard deviation associated with instantaneous R-R variability, SD2 represents the continuous long-term R-R variability [11] and ApEn is often used to understand the regularity of biomedical signals [12]. ApEn shares an 81.1% correlation with LF/HF ratio. Poincaré plots were visually more effective for discriminating autonomic change associated with anesthesia. Ishaque et al. analyzed stress from a VR environment through a Poincaré plot [13]. Villarejo et al. [14] developed a stress sensor based on GSR response, obtained from several stressful and relaxing situations, such as math problems and deep breathing. The device was capable of discriminating between extra effort and divergent states with a 76.56% success rate. There are a wide range of studies describing the correlation between HRV parameters and stress. Standard deviation of R-R intervals (SDRR) reflects the total HRV. It shares a high correlation with LF and total power. It reduces during stressful situations [15,16]. The root mean square of successive differences (RMSSDs) represents the successive differences between neighboring R-R intervals. It shares a high correlation with PNS activity and respiration and reduces in response to stress [17]. The Percentage of successive R-R intervals that differ by more than 50 ms (PNN50) is similar to RMSSDs and shares a high correlation with HF, HRV and respiration. It also decreases due to stress induced [18]. Time domain parameters are considered to be less accurate than frequency domain parameters and non-linear parameters when they involve stress analysis. Unlike the other methods, which can discriminate between whether SNS or PNS activity caused the change in HRV, time domain methods cannot due to the architectural complexity of the R-R intervals [2].

Most effective computing literature perceive and demonstrate the negative impact of video games, which is considered to increase negative emotions, stress and the risk of chronic stress [19]. We present a detailed physiological analysis of stress from subjects after they played a virtual reality (VR) Bubble Bloom game, which was developed by Shaftesbury Technology Inc. (Toronto, ON, Canada), to reduce stress and anxiety. We acquired 3 physiological signals (ECG, GSR and RESP) from 15 subjects, and manually assessed each of them through time, frequency and non-linear methods. It would have been quicker to automatically extract the features using HRV software, such as Kubios HRV, but that often leads to distorted data, which are quite unreliable. Automatic methods use default filters, which do not effectively remove most of the unwanted components from a signal and often produce erroneous data [20]. We manually filtered the signals to achieve the best possible results. We used a statistical *t*-test to verify the significance of the results. We developed a personalized decision tree (DT) CART model through a novel Gini index algortihm to classify binary classes of stress. We dive deeper into stress classification and observe the impact of a novel K-means feature. We further evaluate the model’s performance through a learning curve. It allowed us to gain a better perspective on how well the model fits the data. The contributions demonstrated through this paper are:

1. A comprehensive study which demonstrates the positive impact of a VR Bubble Bloom fish game from Shaftesbury Technology Inc. for stress management. Unlike other studies, we present time, frequency, non-linear, GSR and Respiration analysis of the subjects after they played the game.
2. We developed a personalized DT CART model using a novel Gini index algorithm to classify stress more effectively than other studies.
3. We demonstrate the advantage of feature reduction and present a novel K-means feature developed from 11 other features. This reduced scattered data, magnitude of error and improved model performance at a faster rate.
4. We classified five levels of stress from a VR roller coaster simulation. The purpose of this phase in the experiment was to induce stress through fear and anxiety.

We present various methods used for this study in Section 2 and demonstrate the significance of the proposed methods in Section 3, which describes the results. We compare the performance of our methods to other studies in Section 4, which is the discussion, and conclude our study in Section 5. Overall, this research paper illustrates the importance of both statistical analysis and automatic methods. It illustrates the significance of machine learning for analyzing real-time data, novel faster algorithms and data-driven methods were developed and presented to process real-time data more accurately.

## 2. Materials and Methods

### 2.1. Data Collection

**Figure 2:**
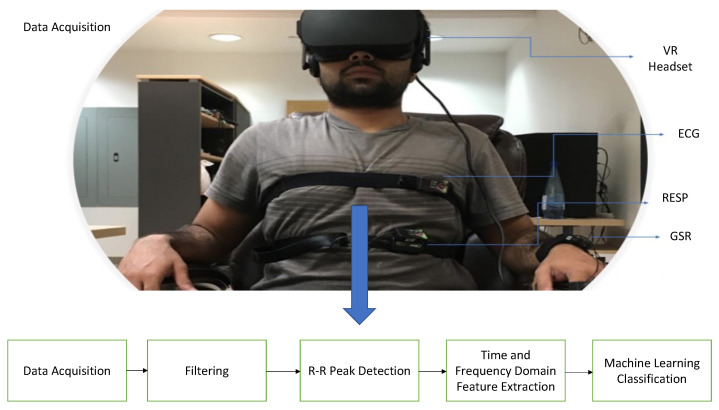
Data acquisition and processing.

Figure 2 illustrates the data acquisition and processing protocol associated with the VR (virtual reality) study in order to understand the impact of the VR Bubble bloom fish game for mitigating stress, as well as classifying it effectively using various signal processing and ML (machine learning) techniques.

**Figure 3:**
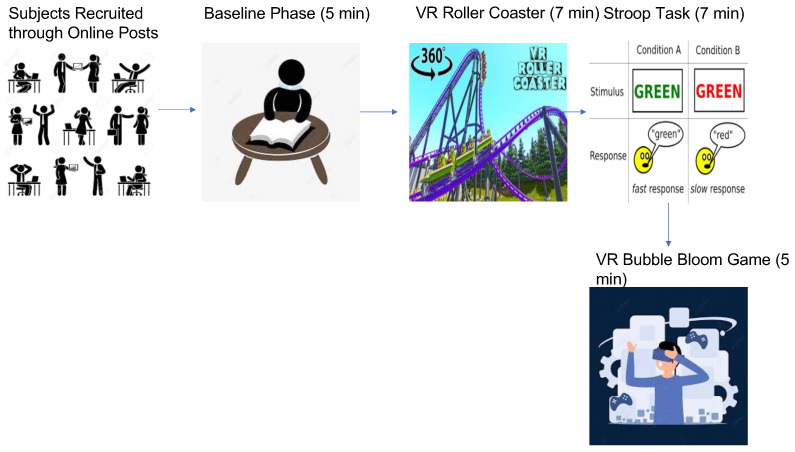
Experiments to obtain data.

Figure 3 illustrates the procedure used to recruit and obtain data from each subject. The data collection protocol for this research was approved by the research ethics board (Grant# 537987-18) of Ryerson University. The dataset was developed from signals obtained from 15 subjects (7 men and 8 women) who were between the ages of 32.4 ± 10.6 years. They were recruited through ads and social media posts in order to assess the impact of VR solutions for comprehending physiological stress due to challenging tasks and the Bubble Bloom fish game. The experiments were conducted at the Ryerson Multimedia Research Laboratory (RML). In order to understand and analyze their underlying physiological function, 3 different physiological signals were measured from each subject for 4 phases, which resulted in 166 signals, which was less than the expected 168 signals but one subject was too stressed to carry on with the roller coaster phase and another signal was not detected due to a technical error associated with wireless transmission of the signal data via Bluetooth. In order to understand HRV, GSR and Respiration associated with stress, 3 physiological signals (ECG, GSR and Respiration) were detected from each subject using a captive T-Sens ECG, T-Sens Respiration and T-Sens Skin Conductance sensors. The experiment consisted of 4 phases: T1, T2, T3 and T4. A notch filter was used to pre-process the signal prior to using signal processing methods to extract the corresponding features. The initial phase was used to measure their baseline/normal parameters by making the subjects read a book or sit still doing nothing. Phases T2 and T3 consisted of a 3D VR roller coaster simulation and cognitive color Stroop game in order to induce stress through negative emotions and cognitive load. The subjects had to observe a VR roller simulation and perform a cognitive Stroop task during the two phases. The last phase utilized the VR Bubble Bloom fishing game from Shaftesbury Technology Inc, where the subjects were instructed to play the 3D VR game. The first phase was 5 min long, whereas the second and third phases were 7 min long, followed by the final phase which was also 5 min long. From the 166 signals (14 subjects × 4 experiments × 3 signals from each − 2 unusable signals), only 150 (11 good subjects × 4 experiments × 3 signals from each + (2 other subjects × 3 good experiments × 3 signals from each)) signals were used to extract the features and develop the final data. There were 11 complete experiments which consisted of 4 phases. Two experiments resulted in signals for the first three phases, as the signals from the last phase could not be properly recorded due to excessive motion artifact associated with the subjects’ movement. Overall, the 50 samples consisted of 13 samples from the baseline phase, 13 samples from the VR roller coaster phase, 13 samples from the Cognitive color Stroop task phase and 11 samples from the VR video game phase. Except for the baseline experiment (T1), other experiments such as the VR roller coaster (T2) and VR video game (T4) included physical movements which were assumed to be activities that were part of the experiments and needed in order to assess the impact of stress due to every aspect of the activities. Table 1 outlines the data collected from each experiment. The game was meant to relax the subjects and return their physiological function back to normal after inducing stress.

### 2.2. HRV, GSR and Respiration Feature Extraction

#### 2.2.1. Preprocessing

The ECG signal was sampled at 250 Hz. It was filtered with a 50 Hz notch filter in order to reduce power line interference, baseline wander and high frequency noise. It does not substantially reduce the oscillations of the signal further above or below the required cut-off frequency. The signal was then smoothed using the wavelet decomposition method using wavedec (db6) with order 10. The GSR signal was pre-processed using a low pass Butterworth filter with an order of 6 and cut-off frequency of 0.5 Hz. The respiration signal was very noisy and was band pass filtered at cut-off frequencies 0.5–35 Hz using a Blackman filter. Both Respiration and GSR signals were sampled at 32 Hz.

#### 2.2.2. Time/Frequency Domain Feature Extraction

In order to measure the ECG parameters from time domain, R-R variation for one R-interval to the next was utilized to assess R-R variability, HRV, nonlinear SD1 (standard deviation of a Poincare plot, which is perpendicular to the ellipse) and SD2 (standard deviation of a Poincare plot, which is along the line of the ellipse), ApEn (approximate entropy, which is used to quantify regularity and uppredictability of fluctuations) and Poincaré plot. The time domain R-R intervals variation was interpolated (cubic interpolation) to smooth the signal, fit the estimates for errors associated and provide an evenly sampled signal. It was then transformed into power spectral density through the autoregressive (AR) (order 10) method and a Lomb–Scargle periodogram (plomb) to delineate the frequency domain variables associated with HRV. In order to discriminate between stressed and relaxed subjects, their physiological parameter was compared with their baseline data. An increase in stress levels often leads to an increase in the LF value, a decrease in the HF value, increased HR, increased LF/HF ratio, reduced HRV and decreased PNN50. Usually, a LF/HF ratio below 1.5 is indicative of a relaxed person, where a ratio above 1.5 illustrates that a person in stressed. However, as stress is associated with a person’s physiological activity, an increase in the LF/HF ratio delineates that a person is in a stressful situation. High stress is correlated to low parasympathetic activity, which is indicated through a decrease in HRV, reduced HF and increased LF. An increase in LF increases sympathetic activity, which increases during stressful situations. It also decreases parasympathetic activity and results in a lower HRV [17]. The LF/HF as a result would increase during stressful situations, resulting in higher sympathetic activity, lower parasympathetic activity and a lower HRV. Similarly, an increase in HR, RESP rate, GSR and decrease in PNN50 have also been associated with increased sympathetic activity, increased LF/HF, an increase in stress, lower parasympathetic activity and a reduced HRV [2]. The increase in respiration rate obtained from the Respiration signal was associated with an increase in stress levels. GSR signal variations/stimulus were analyzed using 30 s windows. It can be used only to indicate stress. Statistical analysis was computed using the *t*-test to test whether the data were significant (indicated by red). A *p*-value > 0.05 rejects the null hypothesis that the data are statistically significant, belong to the same group and there is no significant difference between the data.

#### 2.2.3. K-Means Feature Extraction

K-means is considered as a clustering algorithm capable of grouping data into similar and dissimilar groups [21].

(1) $$minC_{1},\ldots ,C_{k},\mu_{1},\ldots ,\mu_{k}\sum_{i=1}^{k}\sum_{x_{\varepsilon }C_{i}}{∥x-\mu_{i}∥}_{2}$$

where $C_{i}$ represents the cluster extracted from a set of data points *x*, *k* is the number of cluster centers, μ constitutes the set of centers and *i* embodies the average of the data points within the group.

(2) $$\mu_{i}=\sum_{x\epsilon C_{i}}x/n_{i}$$

$n_{i}$ represents the number of samples from the cluster. In order to select the optimal k value, clustering indices are used to assess the standard for grouping. The algorithm to derive the novel 1D K-means feature from multiple features is presented in Algorithm 1. Originally, the K-means algorithm was used as an clustering algorithm, which partitions data based on similar clusters, to discover similarities within the data and predict anomalies without any labels [22]. However, we are the first to convert it into a novel feature which combines the centroids from all the other 11 features, resulting in a comprehensive feature which combines the significant information from every feature.


 **Algorithm 1:** Novel 1D K-means Feature.

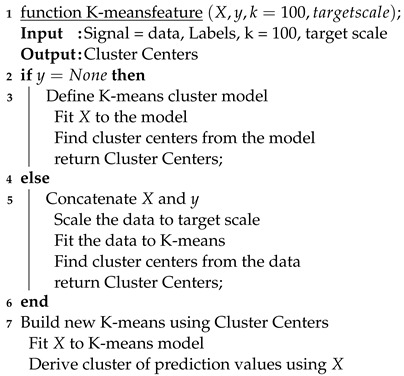




### 2.3. Machine Learning Classification

#### 2.3.1. Binary Classification of Stress

For this experiment, we used ensemble gradient boosting (EGB) a classifier, support vector machine (SVM), naive bayes (NB) and a personalized DT CART model to classify binary classes associated with stress. The dataset consisted of 50 samples obtained from 18 ECG features, which were extracted using time, frequency and non-linear methods. There were 13 samples from the baseline phase, 13 samples from the VR roller coaster simulation phase, 13 samples from the cognitive color Stroop task phase and 11 samples from the VR Bubble Bloom game phase. The HRV features extracted were HR, SDNN, NN50, pNN50, RMSSD, SD1, SD2 and ApEn from the time domain. From the frequency domain, the very low frequency (VLF), LF, HF, total power (TP) and LF/HF ratio were extracted through autoregression (AR) and the Lomb–Scargle periodogram method.

Data Labeling: For this stage of the experiment, we classified data with 5–7 min epochs. Data were manually labeled as relaxed or stressed based on the feature evaluation established in the published literature [2,15,21,23,24]. Eighteen features were analyzed and the sampled data were labeled as 0 to predict a normal subject or 1 to predict a stressed subject, based on their physiological activity. High HR, LF/HF ratio above 3, increased LF, decreased HF, increased ApEN, increased SD1, decreased SD2, decreased pNN50, decreased RMSSD, increased Respiration rate and GSR were commonly labeled as stressed. Normal HR, low LF, high HF, LF/HF ratio below 2, increased pNN50, increased RMSSD, reduced ApEN, reduced SD1, increased SD2 and Resp rate below 25, were indicative of normal/increased HRV were labeled as relaxed. These trends were obtained from the notable literature discussing HRV feature variation associated with stress [25]. Stress is not fixed. It varies based on a person’s reaction and perception towards certain situations [26]. We were able to scrupulously examine the unpredictable behavior of stress and also assess the level of predictablity associated with stress by classifying 5 classes-based phases, which delineates stress as invariable or predefined due to certain circumstances.

EGB: The Ensemble Gradient Boosting classifier is a strong learner which is ideally utilized for regression and classification. The hyper-parameters utilized for classification were a learning rate of 1, n_estimators (represents the number of sequential trees utilized in order to improve performance) of 100 and a max depth of 1 in order to iteratively reduce error function (which is the difference between the predicted and the actual output) and better adapt to the data. By minimizing the error function, the algorithm can obtain the smallest possible error and achieve better classification accuracy, making the prediction akin to the actual output, (which occurs in the future) [27].

SVM: A Support Vector Machine is utilized to find the hyperplane within the N-dimensional space, which can discriminate between the corresponding classes associated with N number of features. We used degree (d = 3) (the degree is responsible for the decision boundary that is used to separate each class, higher degree allows for more flexibility). Gamma was set to scale and the probability was True. A wider margin reduces the generalization error of the classifier.

NB: In a simple model, assuming that the features are conditionally independent, a class of condition density can be represented as a product of one-dimensional densities, which represents the Naive Bayes classification model. The features for this experiment were not independent, making the model resistant to overfitting.

Personalized CART model: This model was developed and personalized in order to classify binary stress by utilizing theories and examples from [28]. Most decision tree models are used to predict labels,

(3) h:X→Y

which understands the transition of feature *X* from its root to leaf node. For binary classification label Y= (0, 1), the nodes are split into right and left nodes based on feature evaluation and threshold. Splitting the nodes into right and left is based on $1_{\left[\begin{array}{c} x_{i}<\Theta \end{array}\right]}$, where $i\epsilon \left[\begin{array}{c} d \end{array}\right]$ represents the feature index and ΘϵR indicates the threshold. A classifier with *Y* = ${\left(0,1\right)}^{d}$ is simply a model with $2^{d}$ leaves and depth d+1, which can result in a lot of nodes. Minimum description length theory is used to reduce redundancy and fit the data appropriately without overfitting. The root node is the beginning of most DT models. It is labeled through the majority voting scheme. The gain measure is used to discriminate the best split and make the best decision.

Novel Gini index: We present a novel Gini index (Algorithm 2), which initially sorts the features and labels prior to splitting the features. Most Gini indexes iteratively go through all the rows in a dataset and calculate the Gini index based on each label and the corresponding features. Our algorithm is more swift and effective in comparison to splitting the data based on a split point ($X_{i}\leq S$), where *i* is the number of features and *S* is the split point. This makes the algorithm much more efficient in comparison to a traditional DT algorithm, which calculates the Gini impurity for each input $X_{i}$ in order to identify the probability of misclassification based on the distribution of classes and corresponding inputs. Our method finds the highest probability of predicting a certain class based on splitting the input into a left or right node and the Gini index associated with each class. The Gini index ultimately makes a decision based on the best split point by iteratively going through all the features and comparing it’s Gini impurity value to a split point. The split point is also calculated by going through all the features and comparing them to a set split point. If it is lower, then it replaces the set point as the new split point. This is much slower than our algorithm because it is calculating that impurity value every time, as well as attempting to find a new split value each and every time it iterates through a feature. Our novel Gini index algorithm initially sorts the features to accurately find all the candidates which are below and above a given split point (we used 0.5). Instead of iterating through all the possible split points, we minimize that to a single logical operation by simply choosing to descend only left or right, instead of either root for numerous decisions, which makes this significantly faster than the original approach [29].
 **Algorithm 2:** Novel Gini index Algorithm.1 function GiniSplit (Features,labels,length(X+y));   **Input**   : features, labels, length(X + y)   **Output**: gini split, cutoff value, length(X + y)2 sorted ← Sort Features  $labels_{total}$ ← y[sorted]  $0_{belowcutoff}$ ← findall0labelsbelowsplitpoint  $1_{belowcutoff}$ ← findall1labelsbelowsplitpoint  $0_{abovecutoff}$ ← totalsumof0labels−$0_{belowcutoff}$  $1_{abovecutoff}$ ← totalsumof1labels−$1_{belowcutoff}$  $gini_{below}$ ← multiplyratiosoftwobelowlabels  $gini_{above}$ ← multiplyratiosoftwoabovelabels  $InitialGini_{split}$ ← add $gini_{above}$ and $gini_{below}$  $Gini_{split}$ ← $\mathrm{find}\;\min\;{\mathrm{gini}}^{’}s\;\mathrm{from}\;$$InitialGini_{split}$  $cutoff_{index}$ ← ${\mathrm{InitialGini}}_{split}\left[Gini_{split}\right]$  cutoff value ← $X[{\mathrm{cutoff}}_{index},length\left(X+y\right)]$ 3 return $Gini_{split}$, cutoff value, length(X+y)

#### 2.3.2. Five Classes in Classification of Stress

We then inspect stress at a deeper level and classify 5 classes of stress from a VR Roller Coaster Simulation. This dataset was developed through the following 11 features: HR, NNRR, AVNN, RMSSD, pNN50, TP, VLF, LF, HF, LF/HF ratio and GSR. Data were collected through wireless Bluetooth sensors and features were extracted in real time from a 30 s window, using the HRV analysis package. Data were generated instantaneously alongside the experiment. It took around 2 min to extract the features from the raw data using 30 s epochs through the HRV analysis package. The dataset consisted of 86 pieces of data. We used similar features throughout all the experiments since they were relevant features developed to study HRV more precisely and provide accurate predictions. We used DT, XGBoost and EGB embedded in a pipeline with min max scaler preprocessor and a Chi-squared test for feature selection and grid search to automatically find the best hyperparameters. They were used to classify relax, low stress, avg stress, mid stress and high stress from a scary VR roller coaster simulation in real time. The features were then transformed to a novel 1D K-means feature, which was also used to classify 5 classes of stress.

Data Labeling: The data were labeled as relax, low, avg, mid and high levels of stress based on the movement of the roller coaster. Each piece of data obtained from 30 s windows were properly labeled from 0–4. Least stressful movements were labeled as 0, due to normal steady roller coaster movements. Dull movements with no loops were labeled as 1. Movements which involved 1 or 2 loops were labeled as 2. Fast activity, multiple variations, continuous loops, twist, turns and underwater dives were labeled as 3. Intense movements, long and continuous loops, scary jumps and scary environment were labeled as 4.

XGBoost: Consists of decision trees which are sequentially added based on the evaluation of the prior one. Unlike other boosting algorithms, each tree can be added in parallel to one and another since they are trained separately. The model was implemented with a pipeline and evaluated using a grid search. The hyperparameters utilized for tuning were n_estimators (n = 100, the number of trees added sequentially to improve the performance of the model), learning rate, max depth, colsample bytree and gamma.

Decision Trees: DTs are supervised learning method used for classification and regression. They are implemented through separation of specific features from a random subset of a K feature within each internal node. Decision trees are very simple and easy to interpret. They present a smart and facile visual representation. The split quality is measured using Gini. The nodes were split using the best splitter.

Pipeline: Both XGBoost and EGB were embedded in a pipeline, along with a minmax scaler preprocessing transformer and Chi-squared test for feature selection. This is a quick and effective method to prepare a dataset with better quality before classification.

Grid Search: It is an effective away to evaluate the model through each combination of hyperparameters. It also indicates the most ideal hyperparameters after classification.

## 3. Results

### 3.1. Physiological Function Associated with Stress

This section evaluates the impact of stress on human physiology through time, frequency and non-linear methods. Three physiological signals were detected from 15 subjects (although 2 subjects had to be excluded from the first three experiments and 4 were excluded from the last one due to the ineffectiveness of the signal since movement can deteriorate signal quality and certain subjects also quit the experiment before finishing) and analyzed in order to gain better insight on stress in response to effective emotions such as fear and anxiety. None of the subjects suffered from any known diseases. Our goal was to assess the impact of the VR reality video to manage stress after stressful activities only.

Although ECG is the most effective, all three signals can be used to effectively analyze HRV. GSR is used to indicate the rate of change associated with electrodermal activity (EDA)/sweat gland activity. The skin conductance response (SCR or phasic component) and skin conductance level (SCL or tonic component) are the two main components of GSR. SCL varies slowly and it is different for each individual. It is uncertain how effective it is for stress analysis or emotional arousal. It is beyond the scope of this study, which is primarily focused on stress analysis through HRV [30,31]. A future study may be conducted to interpret stress through GSR analysis more extensively. SCR can only be used to indicate SNS activity, which increases due to stress, increased body temperature and increased HR. Respiration shares a high correlation with HRV and PNS activity. An increase in the respiration rate above 25 breaths/min is a good indication of increased HR, low HRV, low PNS activity and increased stress. The impact of video games is rarely studied, but this section also demonstrates the impact of a novel VR Bubble Bloom fish game from Shaftesbury Technology Inc. towards stress management. Both GSR and RESP signals can be examined further to analyze stress more extensively but it is beyond the scope of this study, which revolves around stress analysis through HRV.

From power spectral density (PSD) analysis we were able to decipher the LF (0.04–0.15 Hz) and HF (0.15–0.4 Hz) activity during a stressful period. LF increases with increased stress and SNS activity. HF decreases during increased stress and reduced PNS activity. The results were obtained from the baseline phase. The LF value was 846.7110 ms${}^{2}$/Hz. The HF value was 432.6174 ms${}^{2}$/Hz. The LF/HF ratio was 1.9572, which is above 1 but less than 2, indicating the subject was moderately stressed [2]. The right figure resulted in a LF of 2.3397 × $10^{3}$ ms${}^{2}$/Hz, a HF of 2.6527 × $10^{3}$ ms${}^{2}$/Hz and a LF/HF ratio of 0.8820. This was obtained from the same subject during the VR Bubble Bloom game simulation. It indicates the reduction in stress levels, leading to a relaxed individual after the VR Bubble Bloom game simulation.

**Figure 4:**
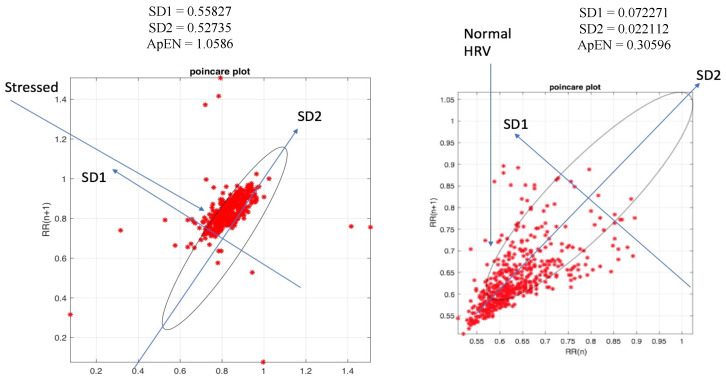
Signal analysis: Poincare plot for stress and normal HRV.

Figure 4 demonstrates the behavior of R-R peaks associated with normal and low HRV through Poincaré plots. It presents a non-linear analysis of HRV, which is typically used to assess HRV associated with cardiovascular diseases such as arrythmia [32] and type 2 diabetes [33]. It shares a 0.811 correlation with LF/HF ratio [10], making it an effective way to measure stress as well. SD1 and SD2 shares a good correlation with SNS and PNS activity. A stressful event resulted in a SD1 value of 0.55827, an SD2 value of 0.52735 and an ApEn of 1.0586. A relaxation phase resulted in SD1 of 0.022112, SD2 of 0.072271 and an ApEn of 0.30596.

Table 2 demonstrates the physiological function and feature values associated with stress. The results effectively demonstrated the positive impact of video games towards stress reduction. The results from T4 (video game phase) indicate that the HR reduced from 76 bpm to 74 bpm, ApEn reduced from 0.875 to 0.757, LF (plomb) reduced from 2.6527 × $10^{3}$ ms${}^{2}$/Hz to 2.3397 × $10^{3}$ ms${}^{2}$/Hz, HF (plomb) increased from 1.49 × $10^{3}$ ms${}^{2}$/Hz to 2.65 × $10^{3}$ ms${}^{2}$/Hz, LF/HF ratio reduced from 1.8019 to 0.882 and GSR_mean reduced from 5.665 to 3.257. A statistical *t*-test was used to evaluate the statistical significance of the features. A *p*-value ≤0.05 allowed us to validate that the feature values from each experiment were statistically significant and reject the null hypothesis that the VR video had no impact on stress reduction. The results evidently exhibit the effectiveness of the video game for increasing PNS activity and stress reduction. Researchers often utilized video games to study facial expressions and human behaviour, but unlike other studies, we use them to study the cause, effect and physiology of stress [34,35].

**Figure 5:**
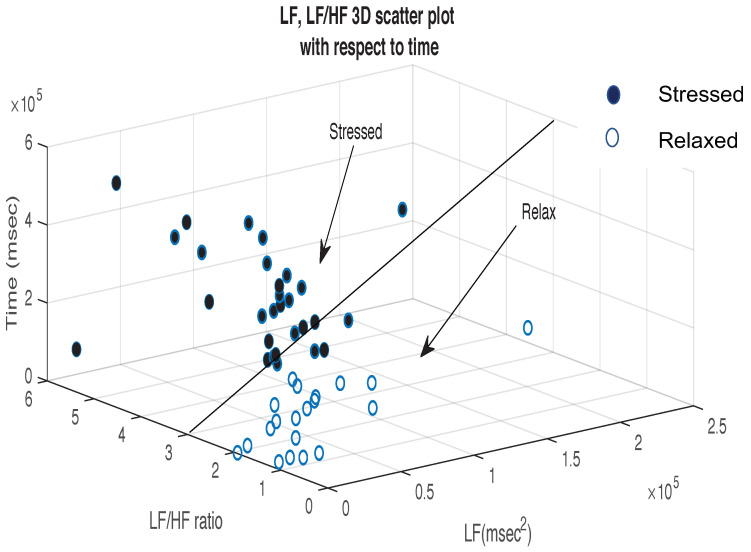
3D scatter plot of LF, LF/HF ratio with respect to time.

Figure 5 demonstrates the LF, LF/HF variations associated with stress with respect to time. Stress analysis is more effective in the frequency domain [2]. An LF/HF ratio of three or less can be considered to be normal. It might be associated with happiness depending on the activity which caused such a response [23]. An LF/HF ratio above three indicates that the subject is suffering from high stress. A high LF is ideally associated with stressed subjects. Evident from the scatter plot, as the experiment shifted from the baseline to the other phases, subjects were more stressed over time.

### 3.2. Model Performance from Binary Classification of Stress

During this phase of thee research we classified binary stress using a strong learner EGB, personalized DT CART model, SVM and NB. The CART model was developed using a novel Gini index in order to classify stress effectively. This section allowed us to explore and validate the efficiency of the personalized CART model in comparison to other established machine learning algorithms. This section also evaluates the effectiveness of the proposed model in comparison to other related studies.

The results from Table 3 illustrate the effectiveness of the personalized DT CART model in comparison to other supervised ML algorithms. The model achieved an accuracy of 87.75%, performing better than complex algorithms such as a SVM and NB, which achieved an accuracy of 60% and 70%, respectively. In order to gain deeper insight about each machine learning algorithm’s performance, make better use of data and obtain more comprehensive information, which would allow us to measure the real performance of each model rather than the best performance, we utilized 5-fold and 10-fold cross-validation. Five-fold cross-validation results in a more efficient use of the data since the data are observed multiple times through multiple training and testing trials, which also allows us to alleviate overfitting. Since we used a smaller dataset (only 50 samples), 5-fold would be the more sensible cross-validation method instead of 10-fold cross-validation in order to reduce the risk of overfitting associated with a large variance. A higher K not only increases the number of folds, it also increases the computational time and variance. The results obtained conspicuously indicate that there is a fluctuation occurring within each training set. Increasing the number of folds causes the algorithm to model the noise associated with the data, which often leads to overfitting. Keeping the size of the dataset in mind, five-fold cross-validation is the most judicious choice, in order to reduce bias and variance but not information, which identifies the relevant relationship between the features and target output. The mean accuracy of the personalized DT algorithm after five-fold cross-validation was 75.77%, with a precision of 74.2% and recall of 74.48%. The performance degraded substantially after 10-fold cross-validation, which resulted in a mean accuracy of 65%, with a precision of 68.33% and recall of 68.33%. This further insinuates that 10-fold cross validation resulted in a high variance and overfitting of the data, which causes a high error rate during the model evaluation. It outperformed the SVM and NB, which resulted in 71.55% (5-fold) and 55.50% (10-fold) and 63.55% (5-fold) and 56.50% (10-fold) accuracies. It is evidently the most potent algorithm ensuing EGB. EGB was the most competent model for classification of stress. It predicted stress with 100% accuracy. It resulted in a mean accuracy, precision and recall of 87.33%, 86.83% and 90.25% from 5-fold cross-validation and 84%, 83.75% and 83.33% following 10-fold cross-validation. It is a gradient boosting model which is capable of learning and adapting to the data, iteratively optimizing the loss function to reduce error and improve performance. EGB is capable of increasing or reducing weights after evaluating the data using the first tree. Depending on the level of complexity associated with data classification, it adds a second tree which can better adapt to the data after learning from the predictions obtained of the first tree. It learns from the data using the loss function

(4) y=ax+b+e,

where *e* is the error associated with the data, *a* and *b* are constant coefficients, *a* is associated with the rate of change and *b* is the *y*-intercept. They allow the model to estimate the relationship between the dependent label *y* and the corresponding independent features *x*. The personalized CART model algorithm is a weak learner which did not utilize a loss function unlike EGB, which was more functional and cogent for data classification.

### 3.3. Classifying Five Classes of Stress from VR Roller Coaster Simulation

During this phase of the research we classified five classes of stress from a VR roller coaster simulation. Label 0 is a relaxed person who is not doing anything stressful or facing a stressful situation. Label 1 indicates low stress, similar to a person whose stress response is triggered due to a surprising or unknown situation. Label 2 is indicative of average stress, which can be induced from a mental task which requires thinking. Label 3 is mild stress, where the subject might be anxious about the time limit of the mental task. Label 5 is high stress, where the subject is in a situation which he can’t adapt to, such as roller coasters, due to the fear associated with riding them. For the experiment, this was determined based on the stages of the task, and how stressful it was, as certain stages were more stressful than others. Initially, 11 features were utilized for classification, the data were scattered and likely to produce a high error rate. In order to reduce errors and irregular distribution of data, the features were then transformed into novel a K-means feature which is derived using the cluster center obtained from the data. The cluster center is the average of all the points that belong to that cluster. By finding the cluster center of the features, we are able to identify the points which shares a close link to all the points, thus significantly reducing the variance associated with random outliers [36].

The models can adapt faster and more efficiently to the K-means feature, which should result in less error and improved accuracy at a faster rate [21].

**Figure 6:**
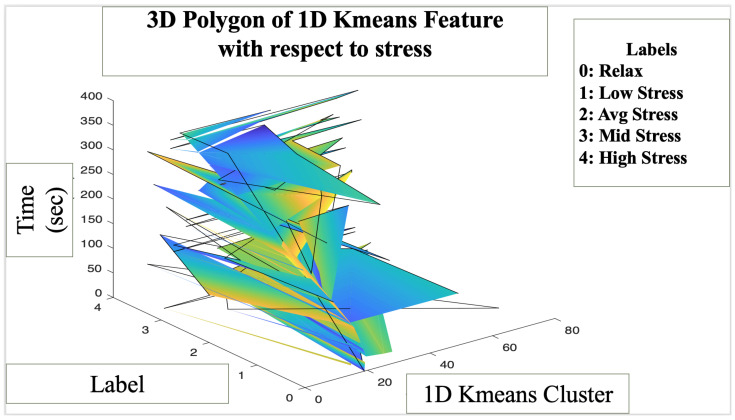
A 3D polygon representation of K-means feature variation due to stress with respect to time.

**Figure 7:**
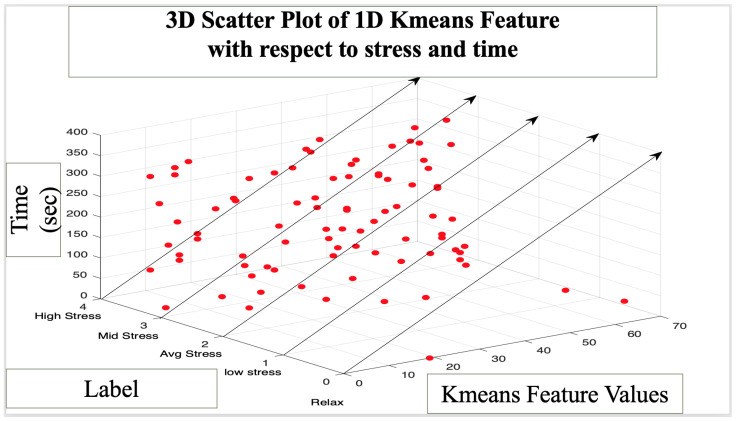
A 3D scatter plot: K-means feature variation due to stress with respect to time.

Figure 6 illustrates a 3D polygon and a 3D scatter plot which describes the feature variation associated with five classes of stress with respect to time. The polygon plot describes the transition of the K-means feature values with respect to the stress classes and time. The color green represents a relaxed label, whereas blue indicates the high stress label, with yellow representing an average stress label. Since it is a 3D graph, the color transition is not as apparent as it would be for 2D or 1D graphs. From the data, we can visually comprehend that the increase in stress levels is not a linear transition. Depending on the phase of the roller coaster simulation, stress induced can range anywhere from low to high. It allowed us to trace stress and interpret its variability with respect to time and the corresponding activity. The major purpose of the roller coaster phase was to induce higher levels of stress prior to the video game phase in order to effectively analyze the impact of the Bubble Bloom game. Figure 7 illustrates a 3D scatter plot which demonstrates the K-means feature variation due to stress levels with respect to time. We were able to comprehend the level of stress due to the phase of the roller coaster. Unlike the polygon plot, scatter plots allowed us to interpret stress more effectively and interpret each point and the level of stress associate based on the phase of the data.

Table 4 displays the model performance obtained from classifying the K-means and all the features. R${}^{2}$ and mean squared error (MSE) performance metrics were used to evaluate the performance of the ML algorithms. Both metrics are very effective for evaluating regression models. MSE measures the average squared error produced by a ML algorithm, where the error is the difference between the predicted value and actual value, which is squared in order to prevent the negative and positive values from canceling each other out. R${}^{2}$ is a statistical method which represents the correlation between the feature values and the regression line. A regression line is a line which delineates the relationship between the features and the corresponding labels. It allowed us to identify how effectively the model adapted to the data and predict the labels. A lower MSE and higher R${}^{2}$ demonstrate a higher level of correlation between ML algorithms and the corresponding dataset [37]. The results allowed us to understand how well the models adapted to the dataset, which consisted of all the features vs. the K-means feature, which inevitably led to less error and a higher accuracy. EGB and XGBoost were embedded in a pipeline and the model’s were evaluated using a grid search, allowing us to tune the models using the most effective hyperparameters. DT was also used. Unlike the other two strong learners, it is the only weak learner. Both EGB and XGBoost were developed through an ensemble of DT. EGB classified five classes with 69.12% accuracy with all the features and K-means feature. However, when using all the features, the performance resulted in a mean squared error of 0.83 and R${}^{2}$ metric of 0.22. In comparison, a mean squared error of 0.22 and R${}^{2}$ metric of 0.83 were achieved after classification through the EGB model using the K-means feature. Classification with the K-means feature resulted in a lower MSE value, which established that feature transformation resulted in less magnitude of error, which was derived from the sum of differences between actual and predicted values. The model also resulted in a higher R${}^{2}$ metric using the K-means feature. Although classification accuracy was the same when using all the features and the K-means feature, the model adapted to the data better upon using the K-means feature. The most optimal hyperparameters for EGB were a learning rate of 0.0001, max depth of 3 and n_estimator of 50 for both classifications. Both DT and XGBoost resulted in 72.2% accuracy, 0.06 mean squared error and 0.72 R${}^{2}$ metric after classification using the K-means feature. The DT model performance was much better using the K-means feature in comparison to when using all the features, which resulted in 50% accuracy, 1.39 mean squared error and 0.04 R${}^{2}$ metric. All the features resulted in more error, less accuracy and worse model performance. XGBoost was able to classify with 67.65% accuracy using all the features, but it also resulted in 0.77 more mean squared error and a 0.5 lower R${}^{2}$ metric. The best hyperparameters were colsample_bytree of 0.1, gamma of 0.1, max_depth of 2, n_estimator of 50 and a learning rate of 1 when using all the features. For the K-means feature, the optimal hyperparameters used were colsample_bytree of 0.1, gamma of 0, max_depth of 4, n_estimator of 150 and a learning rate of 1.

This phase evidently demonstrated the significance of the K-means feature. Pipeline simulation using all 11 features took 2 min and 21 s to complete and classify stress, which was 36 s above the time it took for the pipeline simulation using the K-means feature. All the models performed more effectively and produced fewer errors at a faster rate using the K-means feature. K-means has a training time of O(nkd) and it requires a memory of O(kd), where *d* is the number of features, *n* is the number of data points and *k* is the centroid. For prediction, it needs a time of O(kd) and a memory of O(kd). Most models require more memory and time of O(nd) [21]. The K-means feature which was derived using the K-means algorithm was much faster due to its ability to reduce time and memory consumption associated with more data points and features. It computes the cluster center from each feature within the dataset. Unlike other dimensional reduction methods which eradicate information from most features, it takes all the features into account and is suitable for further algebraic transformation. It can also be utilized as an additional feature to improve model performance.

## 4. Discussion

The main purpose of our research was to analyze the impact of the VR Bubble Bloom fish game from Shaftesbury Technology Inc. towards stress management/reduction, classify binary classes of stress through personalized DT CART model using novel Gini index algorithm and classify five classes using a novel K-means feature. We believe the work presented in this paper is the first such in-depth physiological analysis and classification of stress from video games. We were able to deduce that the video game was an effective activity to reduce stress levels. We are also the first to develop a personalized CART model using a novel Gini index for binary classification of stress. Our algorithm ordered the features, organized the classes based on the split point prior to deriving the Gini index and splitting the data based on Gini, which made the decisions more efficient and accurate. This process allowed our personalized model to process data faster and more effectively than a classical Gini index which calculates the Gini impurity repetitively without tracking the best cut-off and split point to make the most efficient decision. The best accuracy is achieved by a logical decision and not randomly. Most classical models result in accuracy which varies due to the randomness associated with splitting each node. We presented a novel method to transform scattered data into novel K-means, which resulted in less error, better model performance and fit better at a faster rate (required 36 s less to evaluate the data utilized which had 86 samples).

Table 5 exhibits other related studies which utilized physiological features to classify binary and multiple classes of stress. Schimdt et al. [38] presented a novel multimodal dataset which collected ECG, EDA, electromyography (EMG), blood pressure volume (BPV) and RESP data. They analyzed effective emotion at a deeper level than other studies and determined its correlation to stress. They were able to classify binary classes with 93% accuracy using a linear discriminant analysis (LDA) model and three classes of stress with 80% accuracy using the strong learner Adaboost. There are not many effective methods to analyze the correlation between emotion and stress, but Hosseini et al. [39] conducted a study to demonstrate the impact of an emotional recognition system. They acquired RESP, skin conductance (SC), HRV, photoplethysmography (PPG) data and classified emotional stress with 82.7% accuracy using an Elman neural network classifier. Stress develops from the body’s reaction to any physical, emotional or mental changes. Emotional stress physiology is quite different from mental, which is primarily associated with the brain. Saidatul et al. [40] presented an integrated system to detect brain changes associated with mental stress. They analyzed EEG data to understand brain activity associated with mental stress and classified it with 91.7% accuracy through a NN model. Khosrowabadi et al. [41] analyzed chronic mental stress due to exams with a brain computer interface (BCI) and EEG. They were able to classify mental stress with 90% accuracy using a K-nearest neighbors (KNN) algorithm. Few researchers have attempted to study both emotional and mental stress. Sharma et al. [42] analyzed ECG, skin temperature (ST), blood pressure (BP), eye gaze, pupil and EEG data from subjects watching videos. They applied a novel genetic algorithm ensembled with a SVM to classify binary stress with 95% accuracy. Fares et al. [43] analyzed multilevel mental stress using an EEG. They developed a novel error-correcting output codes (ECOC) alogorithm to reduce error and were able to classify mental stress with 94.79% accuracy using a SVM model. Arsalan et al. [44] detected perceived mental stress using an Interaxons EEG headband and developed a novel feature selection algorithm. They were able to classify binary classes with 92.85% accuracy and three classes with 64.28% accuracy using a multilayer perceptron (MLP) model. Our study obtained ECG, GSR and RESP physiological signals from 15 subjects. Although there are not enough subjects to draw conclusions about the general population, we were able to effectively assess our hypothesis and draw a conclusion about the VR video game through research experiments with the limited resources and time available. Gathering enough subjects to comprehend the behavior of the general population would require more resources and time, which could take several years. This experiment was performed quickly within a few months in order to draw possible conclusions, which could be more precise with more assessments. Most studies primarily focused on ML classification and disregarded analyzing the physiology behind stress, but we analyzed the physiological function associated with stress prior to classifying the data in order to understand stress at a deeper level. We utilized XGBoost embedded in a pipeline to clean the data and tune the model with various hyperparameters, allowing us to classify five classes of stress with 72.2% accuracy, using a novel K-means feature. We were able to classify binary stress with 100% accuracy using the EGB algorithm, which outperformed every other related study.

**Figure 8:**
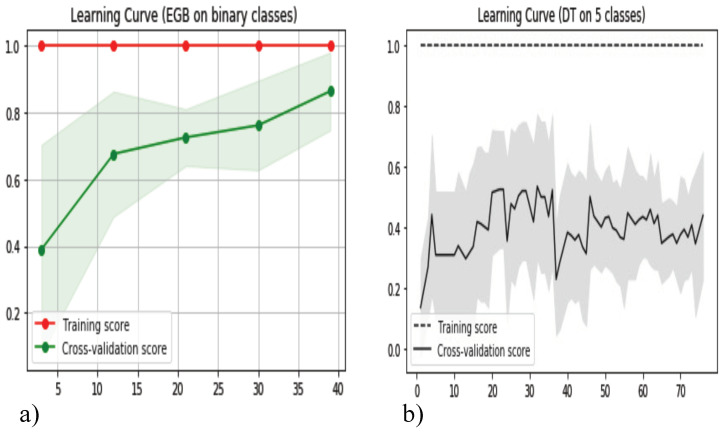
Learning Curve: (**a**) EGB fitted on binary classes dataset (**b**) DT fitted on five classes dataset.

After achieving fairly good accuracy, it was important to understand how well the model fit the data. Both the DT and EGB are flexible models which make it easier to fit/adapt to the data. EGB was developed from an ensemble of DT. It can iteratively optimize the loss function which can reduce error and improve model performance. Both models, however, are susceptible to overfitting. Figure 8 depicts the learning curve obtained from both models for five classes and binary classes classification. From both curves we can distinguish the high variance between the training and validation score, as well as the perfect training score. This is a major limitation which needs to be addressed in the future since a perfect training score is a major indication of overfitting. It indicates that the model learned the noise and random fluctuations as concepts, which does not apply to new data. More data added can hinder and reduce model performance rather than improve it for these types of scenarios. A smaller learning rate, the shrinkage factor and minimizing max depth can significantly reduce the chance of overfitting. Learning curve data analysis is often disregarded, but it is an effective method to better understand model performance and their ability to classify new data with high accuracy.

## 5. Conclusions

The purpose of this paper was to present an in-depth physiological analysis of stress from a VR Bubble Bloom game. The results obtained demonstrated the positive impact of the video game towards stress reduction. We were able to classify binary stress with 87.75% accuracy using a personalized DT CART model and 100% using EGB. We presented a novel K-means feature which resulted in improved model performance. XGBoost embedded in a pipeline was able to classify five classes of stress with 72.2% accuracy using the novel K-means feature as an input. We would like to present a method to automatically classify multiclasses of stress using a cloud server in the future.

## Figures and Tables

**Table 1:** Data collection from each experiment.

Experiments	Number of Subjects	Number of Signals
Baseline Phase	13	39
VR Roller Coaster	13	39
Color Stroop Task	13	39
VR Video Game	11	33

**Table 2:** User 2 Results from (T1) Baseline, (T2) VR Roller Coaster Simulation, (T3) Color Stroop Task, (T4) VR Bubble Bloom Game.

User 2	T1	T2	T3	T4	*p*-Value
Mean HR	84.75	73.41	76.05	74.22	$8.50\times 10^{-5}$
SDNN	0.05	0.07	0.16	0.08	0.03
RMSSD	0.03	0.08	0.21	0.10	0.06
NN50	21	124	148	87	0.04
PNN50	0.20	0.14	0.26	0.25	0.01
SD1	0.02	0.06	0.15	0.07	0.07
SD2	0.07	0.08	0.17	0.09	0.02
ApEN	0.31	0.70	0.88	0.76	0.01
VLF (AR)	12.41	$1.71\times 10^{2}$	$1.72\times 10^{2}$	$1.41\times 10^{2}$	0.01
LF (AR)	3.93	$7.71\times 10^{1}$	140.69	$7.32\times 10^{1}$	0.05
HF (AR)	12.43	$4.89\times 10^{1}$	$2.36\times 10^{2}$	$6.62\times 10^{1}$	0.08
LF/HF (AR)	0.32	1.58	0.60	1.11	0.16
TP (AR)	30.54	$3.25\times 10^{2}$	$5.59\times 10^{2}$	$3.21\times 10^{2}$	0.05
VLF (Lomb)	400.21	$2.08\times 10^{3}$	$2.90\times 10^{3}$	$2.21\times 10^{3}$	0.06
LF (Lomb)	846.71	$3.03\times 10^{3}$	$2.69\times 10^{3}$	$2.34\times 10^{3}$	0.04
HF (Lomb)	432.62	$2.66\times 10^{3}$	$1.49\times 10^{3}$	$2.65\times 10^{3}$	0.02
LF/HF (Lomb)	1.96	1.14	1.80	0.88	0.04
TP (Lomb)	$1.52\times 10^{3}$	$6.14\times 10^{3}$	$5.93\times 10^{3}$	$5.75\times 10^{3}$	0.01
GSR_mean	3.18	6.21	5.67	3.26	0.01
GSR_std	0.04	0.07	0.02	0.04	0.02
RESP (breath/min)	10.22	24.83	24.96	24.35	0.01

**Table 3:** Performance of proposed DT method in comparison to other ML algorithms using 5-fold and 10-fold cross-validation. NB: Naive Bayes, SVM: Support Vector Machine, EGB: Ensemble Gradient Boosting.

ML Algorithms	Accuracy (%)	Precision (%)	Recall (%)
Personalized DT	87.75	90.00	88.00
NB	70.00	63.00	61.00
SVM	60.00	76.00	59.00
EGB	100	100	100
	Acc (5 Folds) (%)	Pre (5 Folds) (%)	Rec (5 Folds) (%)
Personalized DT	75.77	74.20	74.48
NB	63.55	58.95	69.21
SVM	71.55	69.45	62.14
EGB	87.73	86.83	90.25
	Acc (10 Folds) (%)	Pre (10 Folds) (%)	Rec (10 Folds) (%)
Personalized DT	65.00	68.33	68.33
NB	56.50	43.25	51.75
SVM	55.50	33.00	52.50
EGB	84.00	83.75	83.33

**Table 4:** Model evaluation after 5 class classification of stress from VR Roller Coaster Simulation (K-means (*) and all features).

Model	Acc (%)	Mean Squared Error	R^2^
DT *	72.22	0.06	0.72
EGB *	69.12	0.22	0.83
Xgboost *	72.22	0.06	0.04
DT	50.00	1.39	0.04
EGB	69.12	0.83	0.22
Xgboost	67.65	0.83	0.22

**Table 5:** Performance of proposed methods in comparison to other research studies on stress.

Reference	Physiological Signals	Classes	Method	Accuracy
[31]	ECG, EMG	2, 3	LDA, AB	93 (2), 80 (3)
[32]	RESP, PPG	2	Elman Classifier	82.7
[33]	EEG	2	NN, Burg, Yule	91.7
[34]	EEG	2	KNN, SVM	90
[35]	ECG, eye gaze, pupil	2	SVM, ANN	95
[36]	EEG	2	SVM + ECOC	94.79
[37]	EEG	2, 3	MLP, SVM, NB	92.85 (2), 64.28 (3)
[38]	ECG, GSR, RESP	2, 5	Novel DT, GB, XGB	100 (2), 72.2 (5)

## Data Availability

The data were collected at TMU, from 15 subjects. It is not a publicly available dataset.

## Abbreviations

The following abbreviations are used in this manuscript:

DT	Decision Tree
SNS	Sympathetic Nervous Systems
PNS	Parasympathetic Nervous Systems
ECG	Electrocardiogram
GSR	Galvanic Skin Response
RESP	Respiration
VR	Virtual Reality
CART	Classification and Regression Tree
EGB	Ensemble Gradient Boosting
XGB	Extreme Gradient Boosting
HR	Heart Rate
ApEN	Approximate Entropy
LF/HF	High Frequency Ratio
LF	Low Frequency
HF	High Frequency
